# Sporadic cases of lumpy skin disease among cattle in Sharkia province, Egypt: Genetic characterization of lumpy skin disease virus isolates and pathological findings

**DOI:** 10.14202/vetworld.2018.1150-1158

**Published:** 2018-08-23

**Authors:** Fatma M. Abdallah, Hend M. El Damaty, Gamilat F. Kotb

**Affiliations:** 1 Department of Virology, Faculty of Veterinary Medicine, Zagazig University, 44511-Zagazig, Sharkia Province, Egypt; 2 Department of Animal Medicine, Faculty of Veterinary Medicine, Zagazig University, 44511-Zagazig, Sharkia Province, Egypt

**Keywords:** cattle, Egypt, lumpy skin disease, Poxviridae, Sharkia province

## Abstract

**Background and Aim:**

Lumpy skin disease (LSD) is a highly infectious viral disease upsetting cattle, caused by LSD virus (LSDV) within the family *Poxviridae*. Sporadic cases of LSD have been observed in cattle previously vaccinated with the Romanian sheep poxvirus (SPPV) vaccine during the summer of 2016 in Sharkia province, Egypt. The present study was undertaken to perform molecular characterization of LSDV strains which circulated in this period as well as investigate their phylogenetic relatedness with published reference capripoxvirus genome sequences**.**

**Materials and Methods:**

A total of 82 skin nodules, as well as 5 lymph nodes, were collected from suspect LSD cases, and the virus was isolated in embryonated chicken eggs (ECEs). LSD was confirmed by polymerase chain reactions amplification of the partial and full-length sequences of the attachment and G-protein-coupled chemokine receptor (GPCR) genes, respectively, as well as a histopathological examination of the lesions. Molecular characterization of the LSDV isolates was conducted by sequencing the GPCR gene.

**Results:**

Characteristic skin nodules that covered the whole intact skin, as well as lymphadenopathy, were significant clinical signs in all suspected cases. LSDV isolation in ECEs revealed the characteristic focal white pock lesions dispersed on the chorioallantoic membranes. Histopathologic examination showed characteristic eosinophilic intracytoplasmic inclusion bodies within inflammatory cell infiltration. Phylogenetic analysis revealed that the LSDV isolates were clustered together with other African and European LSDV strains. In addition, the LSDV isolates have a unique signature of LSDVs (A11, T12, T34, S99, and P199).

**Conclusion:**

LSDV infections have been detected in cattle previously vaccinated with Romanian SPPV vaccine during the summer of 2016 and making the evaluation of vaccine efficacy under field conditions necessary.

## Introduction

Lumpy skin disease (LSD) is a viral disease of cattle characterized by an appearance of circumscribed firm skin nodules covering all parts of the body with generalized lymphadenopathy [1]. It results in great economic losses due to damage of the skin, reduced milk yield, mastitis, lowered fertility, abortion, and sometimes death due to secondary bacterial infections [2]. Histopathological changes in the skin lesions of LSD are mainly characterized by vasculitis, necrotic epidermis, and eosinophilic intracytoplasmic inclusion bodies [3,4]. LSD is caused by LSD virus (LSDV) which together with sheep poxvirus (SPPV) and goat poxvirus (GTPV) belongs to the genus *Capripoxvirus* (CaPV), subfamily *Chordopoxvirinae*, of the family *Poxviridae* [5]. The CaPV homolog of G-protein-coupled chemokine receptor (GPCR) gene is one of the variable genes within the CaPVs [6] and is a suitable target for genetic differentiation between members of CaPV [7]. Restriction fragment length polymorphism analysis of polymerase chain reaction (PCR) products is also a valuable method for segregating CaPV members [8].

LSD originated in sub-Saharan African countries, in 1929, but has spread to countries of the Middle East [1]. LSD has been spreading from the Middle East to Southeast Europe, affecting EU Member States (Greece and Bulgaria) and several other countries in the Balkans since 2012 [9-13]. In May 1988, an LSD outbreak was confirmed in Egypt after cattle importation from Somalia where it subsequently became endemic [14]. It was reintroduced into Egypt in early 2006 after the importation of cattle from Ethiopia [15]. Later on, sporadic cases of LSD were subsequently reported in Egypt [16-19]. LSD is thought to be transmitted mechanically by biting insects (Aedes aegypti mosquitoes) and Culicoides, and hard Ixodid ticks have also been implicated in the transmission cycle [20-23]. Cattle breeds of both sexes and all ages are susceptible to LSDV, and infection has been observed in wild ruminants such as Arabian oryx and water buffalo [14,24-26].

Diagnosis of LSDV is performed by observation of characteristic clinical signs, virus isolation, electron microscopy, histopathological examination, serological, and molecular techniques [27]. In addition, the suitability of one of the CaPV genes, GPCR gene was described by Cao *et al*. [28] for host range phylogenetic grouping of CaPVs [7].

To date, many vaccines have been tried to control LSD in cattle using strains of CaPV derived from sheep or goats [29]. The attenuated SPPV vaccines, such as KSGPO-240 and Romanian SPPV strains, have been used against LSDV [30,31]; however, partial protection has been recorded in cattle vaccinated with these vaccines [32,33].

The aim of this study was to investigate the sporadic cases of LSD that appeared in Egypt during the summer of 2016 in vaccinated cattle using virus isolation, molecular characterization, and histopathological examination.

## Materials and Methods

### Ethical approval

The Animal Ethical Committee of the Faculty of Veterinary Medicine, Zagazig University, Egypt, approved the present study.

### Animal selection

The cattle farms under investigation were selected based on the presence of clinical signs consistent with LSD during the period of spanning July-December 2016 in Sharkia province, Egypt. These farms were visited, and all suspect cases were clinically examined according to Radostits *et al*. [34]. The cattle in the farms under investigation were previously vaccinated with sheep pox, Romanian strain (10^3.5^ TCID 50/dose) in March 2016.

### Sample collection and preparation

Skin nodules (n=82) were aseptically collected through surgical excision under local anesthesia from 82 animals (65 adult cattle and 17 calves) suspected to have LSD. A total of 5 lymph nodes were collected from dead calves in addition. The samples were transported in phosphate buffer saline (PBS) with 10% antibiotic solution for the laboratory. Each sample from each clinical case was ground using sterile mortar and pestle, suspended in PBS containing 10% antibiotic solution. Each tissue homogenate was centrifuged at 3000 rpm for 10 min at 4°C. The clear supernatant fluid of each tissue homogenate was frozen at −70°C for virus isolation [35].

### Virus isolation into embryonated chicken eggs (ECEs)

The virus isolation was carried out into 11-day-old ECEs from commercial non-vaccinated chickens through the chorioallantoic membrane (CAM) route [36]. Briefly, 200 µl from supernatant fluid of each tissue homogenate was inoculated into five ECEs, incubated for 5 days at 37°C and then examined daily for characteristic pock lesions on infected CAMs. The infected CAMs were suspended in PBS, minced using pestle and mortar, and then centrifuged at 3000 rpm for 10 min at cooling centrifuge. The supernatant fluid of CAMs was kept at −20°C for viral DNA extraction.

### Histopathological examination

The collected skin nodules and lymph nodes were fixed in 10% buffered neutral formalin and embedded in paraffin. 5 µ thick paraffin sections were stained with hematoxylin and eosin [37] and then examined microscopically.

### Viral DNA extraction

Viral DNA was extracted from infected CAMs using Gene JET^™^ Genomic DNA Purification Kit (Fermentas) according to the manufacturer’s instructions. Commercially available and lyophilized sheep pox vaccine virus (Romanian strain) was used as positive control, and negative control was included in each reaction.

### PCR amplification

Oligonucleotide primers designed by Le Goff *et al*. [7] and Ireland and Binepal [38] were used as follows: Forward primer, 5’-TTTCCTGATTTTTCTTACTAT-3’ and reverseprimer,5’-AAATTATATACGTAAATAAC-3’ for amplification of a specific fragment of the attachment gene (192-bp) and two primers (5’-TTAAGTAAAGCATAACTCCAACAAAA ATG-3’ and 5’-TTTTTTTATTTTTTATCCAATGCTA ATACT-3’) for amplification of the full-length GPCR gene of CaPVs (metabion International AG Company, Germany). Two additional primers (5’-GATGAGTATTGATAGATACCTAGCTGTA GTT-3’ and 5’-TGAGACAATCCA AACCACCAT-3’) were positioned internally for sequencing of 6961-8095 nucleotide sequences of the GPCR gene [7]. Amplification was done under the following conditions: Initial denaturation cycle at 95°C for 2 min, 40 cycles (denaturation at 95°C for 30 s, annealing at 55°C for 30 s, and extension at 72°C for 1 min), followed by a final extension cycle at 72°C for 10 min using Dream Taq™ Green PCR Master Mix (Fermentas). Negative and positive controls were included in each reaction. 5 µl of amplified amplicons were separated on 1.5% ethidium bromide-stained agarose gel electrophoresis at 100 V for 30 min. The amplified amplicons were visualized in comparison with gene Ruler 1 kb Plus DNA Ladder (Thermo Scientific™) using ultraviolet light transilluminator.

### Sequencing of GPCR gene

The obtained fragments of GPCR gene were purified using Gene JET™ genomic PCR purification kit (Fermentas) according to the manufacturer’s instructions. Each purified PCR product was sequenced in both directions using an automated sequencer (ABI 3730XL machine; Macrogen Inc., Korea).

### Sequence alignment and phylogenetic analysis

The GPCR gene nucleotide sequences were compared and aligned with those of reference virulent and vaccine CaPV strains in the GenBank (Table-1). Comparative alignment of the GPCR gene sequences was performed using the ClustalW Multiple alignment of BioEdit Version 7.0 software [39]. Sequence identities and divergences were computed utilizing MegAlign software (DNA STAR^®^ Lasergene^®^ version 7.2, USA). A phylogenetic tree was constructed for the GPCR gene nucleotide sequences using the neighbor-joining tree method employing the Kimura 2-parameter model in MEGA6.06 software with 1000 bootstrap replicates [40].

**Table-1:** Details of the isolated LSDV and CaPV reference strains retrieved from GenBank whose GPCR sequence was analyzed and compared in the current study.

Isolates	Country of isolation	Year of isolation	GenBank accession number
LSDV_Egypt/2016-01	Egypt	2016	MG970343
LSDV_Egypt/2016-02	Egypt	2016	MG970344
LSDV_Egypt/2016-03	Egypt	2016	MG970345
LSDV_Egypt/2016-04	Egypt	2016	MG970346
LSDV_Egypt/2016-05	Egypt	2016	MG970347
LSDV18( Egypt/89 Ismailia)	Egypt	1989	FJ869377
Evros/GR/15	Greece	2015	KY829023
Egy_water buffalo (Buffalo LSDV Egypt/Mansoura 11)	Egypt	2011	KP071937
Egy_cattle (Cattle LSDV Egypt/Mansoura 11)	Egypt	2011	KP071936
SERBIA/Bujanovac/2016-	Serbia	2016	KY702007
Adama/B02/2011	Ethiopia	2011	KP663691
LSDV8 (LSDV vaccine Nigeria)	Nigeria	-------	FJ869368
LSDV9 (Sudan/06 Obeid)	Sudan	2006	FJ869369
LSDV TURKEY/201501	Turkey	2015	KR024779
Wenji/B03/2011	Ethiopia	2011	KP663710
SPPV11(Romanian strain)	Morocco	-------	FJ869378
GTPV13 (Turkey/98 Denizli)	Turkey	1998	FJ869356
GTPV14 (Iraq/61 Gorgan)	Iraq	1961	FJ869357
GTPV12 (Oman/84)	Oman	1984	FJ869359
GTPV4 (Nigeria/99)	Nigeria	1999	FJ869364
GTPV10 (Bangladesh/86)	Bangladesh	1986	FJ869355
GTPV15 (Sudan isolate)	Sudan	-------	FJ869361
GTPV8 (Saudi Arabia/93)	Saudi Arabia	1993	FJ869360
SPPV14 (Nigeria/99)	Nigeria	1999	FJ869381
SPPV15 (KS1)	Kenya	-------	KJ818283
SPPV23 (Algeria/05 Illizi)	Algeria	2005	FJ869386
SPPV13 (Senegal Sangalcam/88)	Senegal	1988	FJ869380
SPPV9 (Tunisia/01 4P2)	Tunisia	2001	FJ869350
SPPV21 (Turkey/98 Darica)	Turkey	1998	FJ869383
SSPV8 (Tunisia/01 3P3)	Tunisia	2001	FJ869349
SPPV7 (Tunisia/01 23P2)	Tunisia	2001	FJ869348
SPPV24 (Oman/84)	Oman	1984	FJ869390

LSDV=Lumpy skin disease virus, CaPV=Capripoxvirus, GPCR=Gproteincoupled chemokine receptor, SPPV=Sheep poxvirus

## Results

### Clinical cases

**Figure-1:**
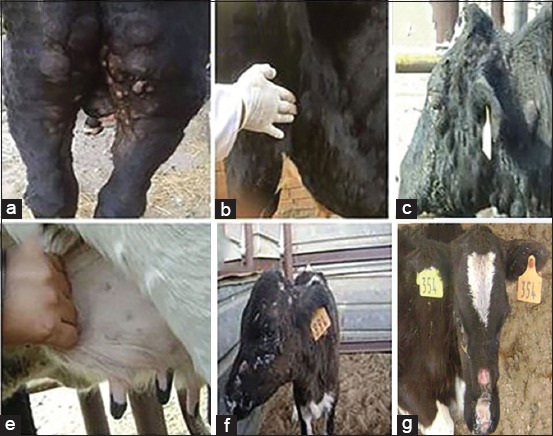
Cattle presenting clinical signs of lumpy skin disease (LSD) in Egypt, 2016. (a and c) Characteristic large firm skin nodules were distributed all over the body, (b) enlarged prefemoral lymph node, (d) small circumscribed dermal nodules (miliary form) on the udder, (e) necrotic skin nodules and ulceration all over the body of a calf, (f) a sitfast lesion developed on the skin of a calf infected with LSD virus after central area of skin nodule was indurated.

All suspected cases were clinically examined for the presence of LSD lesions. The most significant clinical signs were the presence of characteristic skin nodules which covered the entire skin surfaces of the affected cattle including head, neck, trunk, perineum, udder, and teats (Figure-1a-d). The skin nodules involved the epidermis, dermis, subcutaneous tissue, and the musculature. Necrotic nodules and ulcerations were also seen in the affected animals (Figure-1e). Most nodules were necrotic with the formation of deep scabs called sitfast (Figure-1f). In addition, the affected animals showed several complications such as depression, inappetence, salivation, naso ocular discharge, pneumonia, corneal opacity, mastitis, and later on inability to rise and stand on their own (recumbent animals) (Figure-1f). Lymphadenopathy could be demonstrated, especially of the prefemoral (Figure-1b) and prescapular lymph nodes.

### Virus isolation

Positive LSDV isolations characterized by focal white pock lesions on the CAM of the ECE, with thickening after either the first or second passage, were made. In addition, the membranes were hemorrhagic and congested.

### Histopathology

**Figure-2:**
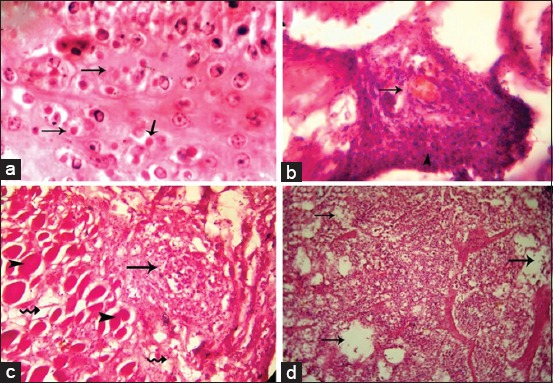
Microscopic examination of skin lesions and lymph nodes in lumpy skin disease virus affected cattle. (a) Eosinophilic intracytoplasmic inclusion bodies (arrows). (b) Necrotic vasculitis in dermal arteriole with infiltration of neutrophils (arrow). (c) Zenker’s necrosis in the dermal muscles (arrowheads) and mononuclear cells aggregation (arrow). (d) Severe edema (arrows) and infiltration of neutrophils.

Histopathological examination of the skin nodules revealed some ballooning degeneration, microvesicles formation, and eosinophilic intracytoplasmic inclusion bodies in the intact epidermis (Figure-2a). Almost all of the dermal blood vessel walls were thickened, necrotic, and diffusely infiltrated with inflammatory cells (necrotic vasculitis) (Figure-2b). The necrosis and the infiltrates extended to the perivascular and subcutaneous tissue in addition to Zenker’s necrosis in dermal muscles (Figure-2c). Histopathological examination of the lymph nodes showed severe edema and neutrophils infiltrations (Figure-2d).

### Detection of the attachment and GPCR genes of CaPVs by conventional PCR

Conventional PCR was performed on infected CAMs as well as commercially available live attenuated SPPV vaccine (Romanian strain). Two PCR reactions were performed for confirmatory identification of LSDV isolates through partial amplification of the attachment gene (192-bp) and host range grouping of CaPVs through full-length amplification of the GPCR gene (1158-bp). DNA sequencing of the GPCR gene fragments was carried out for five isolates named LSDV_Egypt/2016-01, LSDV_Egypt/2016-02, LSDV_Egypt/2016-03, LSDV_Egypt/2016-04, and LSDV_Egypt/2016-05. The GPCR gene nucleotide sequences were submitted to the GenBank database under accession numbers (MG970343-MG970347) (Table-1).

### Sequence analysis of LSDV GPCR gene

**Figure-3:**
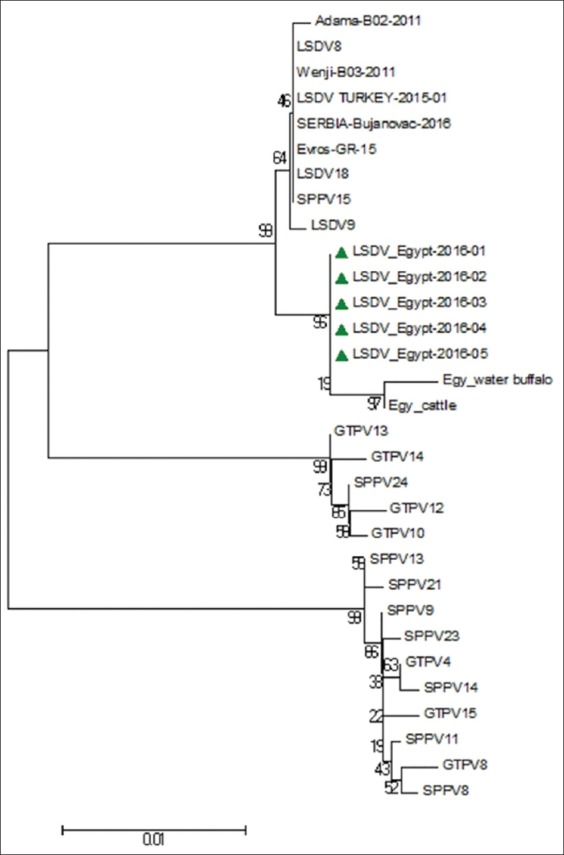
Neighbor-joining (N-J) tree depicting phylogenetic relationships of the lumpy skin disease viruses isolated in this study and other capripoxvirus isolates based on G-protein-coupled chemokine receptor nucleotides sequences. The tree was analyzed by N-J analysis with 1000 bootstrap replicates.

**Figure-4:**
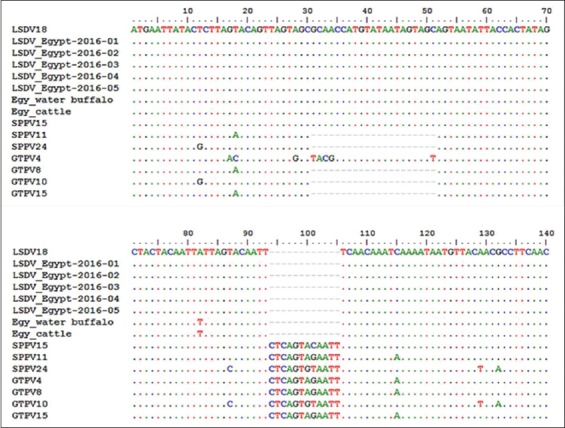
Alignments of the first 140 nucleotides of the G-protein-coupled chemokine receptor gene of lumpy skin disease virus (LSDV) isolates and those of other capripoxvirus recovered from GeneBank. Addition of 21 nucleotides from position 31 to 51 and deletion of 12 nucleotides from position 94 to 105 are evident in the sequences of the newly isolated LSDV when compared with those of goat poxvirus and sheep poxvirus.

**Figure-5:**
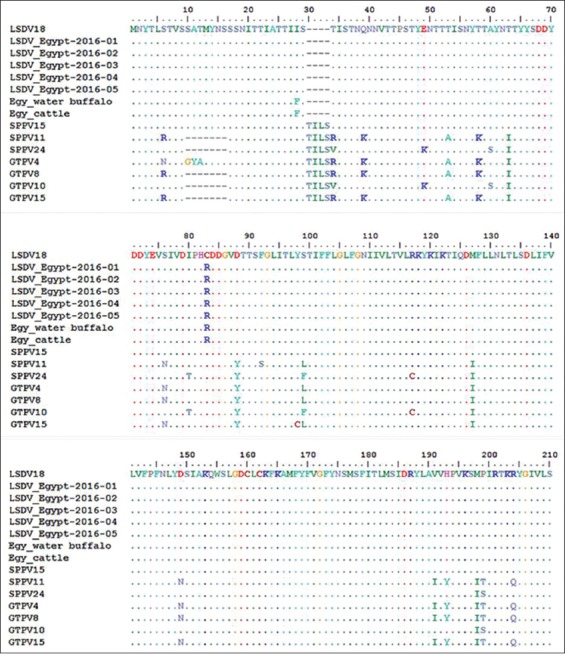
Alignment of the first 200 deduced amino acids of the G-protein-coupled chemokine receptor gene of the isolated lumpy skin disease virus (LSDV) isolates with those of capripoxvirus retrieved from GeneBank, showing the unique signature of LSDV (A11, T12, T34, S99, and P199).

The nucleotide sequences of the GPCR gene of five LSDV isolates aligned with those obtained from GeneBank (Table-1) and showed that the five LSDV isolates were 100% identical with each other. The isolated LSDV showed high nucleotide sequence homology with virulent strains from different regions such as LSDV18, Evros/GR/15, SERBIA/Bujanovac/2016, Adama/B02/2011, LSDV9, LSDV TURKEY/2015-01, Wenji/B03/2011 and Buffalo LSDV Egypt/Mansoura 11, and cattle LSDV Egypt/Mansoura 11 and vaccine strains (LSDV8 and SPPV15), which ranged from 99.3% to 99.7%. However, GPCR gene nucleotide homology was 93.6% between the field viruses isolated in this study and the vaccine strain (Romanian SPPV strain [SPPV11]) used to prevent LSD in the cattle herds under study. The phylogenetic tree of CaPVs based on the alignment of the nucleotide sequences (6961-8095) of the GPCR gene (Figure-3) showed that the LSDV isolates were segregated into the LSDV cluster and were genetically closest to Buffalo LSDV Egypt/Mansoura 11 and cattle LSDV Egypt/Mansoura 11 with a percentage of 99.4% and 99.7%, respectively [41]. In addition, the phylogenetic analysis based on GPCR nucleotides sequences did not reveal differences between the vaccine and virulent strains of LSDV, SPPV, and GTPV. Furthermore, SPPV15 (strain KS-1) which was originally isolated from a sheep had a specific LSDV signature and integrated within the LSD strain cluster [42]. Nucleotide sequences of GPCR gene of the LSDV isolates showed a 21 nucleotide insertion and a 12 nucleotide deletion within the five isolates when compared with published sequences of LSDVs in GeneBank (Figure-4). The deduced amino acid sequences of the GPCR gene revealed the absence of (T30, I31, L32, and S33) amino acid residues and gave the new isolates a specific and unique signature among LSDVs (A11, T12, T34, S99, and P199) (Figure-5).

## Discussion

LSDV is a highly contagious viral disease of cattle causing substantial economic losses to the cattle industry. LSD has been reported in several regions of Africa and the Middle East, including Egypt, Israel, Iraq, Iran, Lebanon, and Jordan. Recently, it was transmitted to Europe involving Turkey, Cyprus, and Greece. Sporadic outbreaks of LSD have occurred in Egypt after LSD was first formally recorded in Ismailia province since 1988. The objective of this study was to determine the phylogenetic relatedness of LSDV which caused the recently observed sporadic cases of the disease among cattle in Egyptian farms during the summer of 2016 with other CaPVs whose sequences are published in GenBank and describe the pathological changes in the clinically affected cattle.

Multiple skin nodules are the characteristic features of LSD, which may have inverted conical necrosis (sitfast) with enlargement of superficial lymph nodes. Along with, histopathological examination of the skin nodules revealed eosinophilic intracytoplasmic inclusion bodies. The clinical symptoms and histopathological changes reported in the present study were consistent with the LSD lesions recorded in previous outbreaks [43,44].

In this study, conventional PCR was used for confirmation of LSDV where viral DNA was detected in the isolates using two sets of primers specific to the partial sequence of the attachment gene (192-bp) and the full-length GPCR gene (1158-bp). The results are compatible with previous reports which concluded that the PCR could be used in the identification of LSDV in biopsy samples, tissue culture, skin, blood samples, semen, and infected CAMs of ECEs [26,45-47].

The GPCR gene of the LSDV was amplified by PCR, and the correct amplicon size (1158 bp) obtained. Sequencing of the amplicons revealed an open reading frame of 1134bp. Phylogenetic analysis of GPCR gene sequences showed that LSDVs of the summer of 2016 are grouped with LSDV isolates from Kenya, Nigeria, Ethiopia, Sudan, Turkey, Serbia, and Greece (Figure-3). In addition, the phylogenetic analysis confirmed that CaPVs could be divided into three distinct clusters as previously described using full genome sequence comparison [48].

GPCR gene nucleotide sequence comparisons revealed a high level of homology (99.3%-99.7%) between circulating isolates in the present study and referenced LSDV isolates from different regions in Africa, Middle East, and Europe, suggesting that LSDVs do not undergo rapid genetic alterations based on GPCR gene sequences [7]. Furthermore, the deduced amino acids sequences of GPCR genes of cattle LSDV isolates confirmed that they have the unique signature of LSDVs previously reported by Le Goff *et al*. [7], who stated that amino acids (A11, T12, T34, S99, and P199) are the unique signature of LSDVs.

The control and prevention of LSDV depend on vaccination, movement restrictions, and vector control [9,15,49]. LSD has been observed in the cattle of this study regardless of previous vaccination using the Romanian SPPV vaccine. In addition, a similar phenomenon has been reported in Ethiopia and Oman [15,44]. This lack of protection may be attributed to the lack of cross-protection of SPPV vaccine strains against circulating virulent field LSDV strains and/or SPP, GTP, and LSD overlap, causing clinical diseases in vaccinated cattle [50]. In Egypt, commercially available vaccines as Romanian SPPV vaccine and Kenyan SPP and GTP virus vaccine (KSGP virus O-240 and O-180 strains) have been used for immunization the cattle against LSDV [1,33]. Hence, it is necessary to evaluate the efficacy of those vaccines under field conditions as well as measure the immune responses, followed by an experimental challenge in a controlled environment.

## Conclusion

LSDV infections have been detected in cattle previously vaccinated with Romanian SPPV vaccine during the summer of 2016 and evaluation of vaccine efficacy under field conditions is warranted.

## Authors’ Contributions

FMA, HME, and GFK planned the study design and analyzed the data. HME collected the samples for virus isolation and histopathological examination. FMA and GFK carried out the laboratory work. FMA and HME drafted and reviewed the manuscript. All authors read and approved the final manuscript.
